# From multidimensional neuropsychological outcomes to a cognitive complication rate: The International Subarachnoid Aneurysm Trial

**DOI:** 10.1186/1745-6215-9-13

**Published:** 2008-03-14

**Authors:** Richard B Scott, Fiona Eccles, Andrew Lloyd, Katherine Carpenter

**Affiliations:** 1 Russell Cairns Unit, John Radcliffe Hospital, Oxford, UK; 2 Oxford Outcomes, Oxford, UK

## Abstract

**Background:**

The neuropsychological arm of the International Subarachnoid Aneurysm Trial (N-ISAT) evaluated the cognitive outcome of 573 patients at 12 months following subarachnoid haemorrhage (SAH). The assessment included 29 psychometric measures, yielding a substantial and complex body of data. We have explored alternative and optimal methodologies for analysing and summarising these data to enable the estimation of a cognitive complication rate (CCR). Any differences in cognitive outcome between the two arms of the trial are not however reported here.

**Methods:**

All individual test scores were transformed into z-scores and a 5^th ^percentile cut-off for impairment was established. A principal components analysis (PCA) was applied to these data to mathematically transform correlated test scores into a smaller number of uncorrelated principal components, or cognitive 'domains'. These domains formed the basis for grouping and weighting individual patients' impaired scores on individual measures. In order to increase the sample size, a series of methods for handling missing data were applied.

**Results:**

We estimated a 34.1% CCR in all those patients seen face-to-face, rising to 37.4% CCR with the inclusion of patients who were unable to attend assessment for reason related to the index SAH. This group demonstrated significantly more self and carer/relative rated disability on a Health Related Quality of Life questionnaire, than patients classified as having no functionally significant cognitive deficits.

**Conclusion:**

Evaluating neuropsychological outcome in a large RCT involves unique methodological and organizational challenges. We have demonstrated how these problems may be addressed by re-classifying interval data from 29 measures into a dichotomous CCR. We have presented a 'sliding scale' of undifferentiated individual cognitive impairments, and then on the basis of PCA-derived cognitive 'domains', included consideration of the distribution of impairments in these terms. In order to maximize sample size we have suggested ways for patients who did not complete the entire protocol to be included in the overall CCR.

**ISAT trial registration:**

ISRCTN49866681

## Background

The International Subarachnoid Aneurysm Trial (ISAT) is the largest ever randomized trial in the treatment of subarachnoid haemorrhage (SAH). Clinical outcomes have been reported at 2 months and 1 year, following neurosurgical clipping or endovascular coiling as a treatment for ruptured intracranial aneurysm [1,2]. The primary outcome was the proportion of patients with Modified Rankin Scale (mRS) grades 3–6 (i.e., dependency or death). ISAT presented an opportunity to explore 'secondary' neuropsychological outcomes following these interventions, which was exploited by assessing patients randomized into ISAT across 8 UK centres, who were surviving 12 months after treatment [3]. This sample represented a UK sub-group of the main ISAT trial and will be referred to as N-ISAT.

N-ISAT represents a clinical dataset that is unique in its size and complexity. Trained examiners administered a neuropsychological battery to 573 patients; other clinical interview and quality of life questionnaire data were also obtained. Other than the results from one questionnaire, these data are not reported here. The test battery needed to sample a range of intelligent behaviours with sufficient sensitivity to detect the quality of impairments that might be associated with a heterogeneous distribution of SAH lesions; this involved 29 measures drawn from 21 tests. A detailed battery was preferred to enable the exploration of more selective brain-behaviour relationships, and importantly, to minimize the potential for type 2 audit errors. For example, a battery of insufficient range and sensitivity might fail to identify potentially disabling deficits in executive skills. However, a comprehensive range of neuropsychological tests scores is far from ideal from the point of view of communicating outcomes parsimoniously to neuropsychologically naïve trial, medical or patient audiences.

Cognitive outcomes are routinely reported by employing parametric inferential statistics. For example a multivariate procedure comparing two arms of a randomised trial across a range of tests to investigate where preferential cognitive outcomes lie. However even if *consistent *group differences are identified across a test battery in favour of one or other trial arm, these would be small, (in terms of raw scores) revealing little about their clinical significance or meaning, and nothing about the number of clinically impaired individual outcomes. A more significant difficulty with this approach is that cognitive outcomes appear to be non-orthogonal in relation to outcomes as measured in terms of handicap scales [4]. Thus, if secondary cognitive outcomes are to be mapped meaningfully onto primary trial outcomes that have already been 'dichotomized' (i.e., in terms of the proportion of dependent or dead, as opposed to alive independent patients), then cognitive outcomes must also be classified into 'handicapped' versus 'non-handicapped' individuals. Only then would it be possible to determine any differences between the two arms of a trial in terms of the degree of any 'additional' cognitive disability.

The task of identifying individuals with a cognitive 'complication' (i.e., a classification of 'significant cognitive impairment') involves two key challenges. The first is determining where to place an appropriate 'cut-off' on individual test scores, (i.e., below which a performance can be said to be 'significantly' impaired). A score may for example be judged relative to that individual's estimated pre-morbid state, a normal control population, or an equivalent clinical population, with each perspective having both advantages and disadvantages. The second challenge is concerned with determining how multiple test scores are to be summarized or distilled down into a smaller number of cognitive constructs or 'domains'. Thus for example a particular constellation of impaired tests scores might be said to represent an impairment in language, spatial memory or executive skills. An individual might be impaired in one or more of these domains (with more or less consistency across tests), and a determination would need to be made about whether that crossed the threshold of functional or clinical significance.

The selection of an optimum number of cognitive domains to encapsulate a data set, and the allocation of individual tests to these domains, are tasks that can be achieved simultaneously and empirically by a principal components analysis (PCA). A PCA aims to mathematically transform (possibly) correlated test scores into a smaller number of uncorrelated principal components or underlying 'factors', while retaining most of the original variability in the data set. Examination of the distribution of tests, and their loadings, represented in the underlying structure (i.e., the 'factors') of the data set, should enable the mapping of factors onto recognizable cognitive 'domains'. A drawback of this approach is that the patterns of shared variance, though empirically-derived, reflect the neuropsychological characteristics of the index clinical population rather than a normal control group [5]. The cognitive domains derived from a PCA would therefore be essentially un-reproducible. An alternative approach is to postulate a priori, or on the basis of explicit criteria, a number of cognitive 'domains' purported to be sampled by selected tests in the battery; a 'composite' domain score can then be obtained by summarizing the relevant individual test scores. This approach is reproducible and allows for the referencing of individuals' test scores against normal (or individual pre-morbid) control data, however the selection of domains and the tests that comprise them, may be criticized as being relatively arbitrary.

This paper explores and presents explicitly, these and other methods that were applied to the task of estimating a cognitive complication rate (CCR) for the N-ISAT data set. The reduction of sensitive interval data yielded by a battery of neuropsychometric tests down into binary outcomes is a retrograde step from the point of view of the richness of the data and the dangers of dichotomisation. However this is what routinely occurs in clinical neuropsychological practise, where the results of various investigations and presenting factors are weighed and an 'impaired' versus a 'not impaired' judgement is made. With no available or agreed method, benchmark, test or scale for determining a 'cognitively impaired' classification, the 'gold standard' is, by default, an expert neuropsychological opinion. With this measure unavailable in a large multi-centre RCT, it follows that, in the first instance, there is no external validation criterion available check the reliability or validity of classifications based upon a numerical algorithm. The task here is therefore to match and mirror the process of expert clinical neuropsychological classification in our analysis of the N-ISAT data set as systematically and transparently as possible. By illustrating the consequences (i.e., for an estimated CCR) of various assumptions and techniques of analysis, we would hope to accommodate a range of clinical opinion and expose our methods to constructive criticism but at the same time, work toward developing a reproducible heuristic that may be of assistance in similar studies.

## Methods

### Patients and design

Multicentre ethical approval was given by Oxford REC A (reference number: 98/5/73). All patients gave informed consent. In May 2002, recruitment to ISAT was closed. At this time point 836 patients had been enrolled in the 8 UK centers participating in N-ISAT (between June 1997 and November 2001) of whom 771 were alive at 12 months. This 'available' sample of 771 was invited to attend neuropsychological follow-up. Of the 713 who could be contacted, 573 were eventually seen for face-to-face assessment.

### Cognitive assessments

The N-ISAT assessment battery was designed to sample cognitive function comprehensively; it has been described and referenced in detail elsewhere [3]. There were 29 measures drawn from 21 psychometric tests. For the purposes of accommodating a range of patient disability and disposition, the test battery was organized a priori into 'screening', 'core' and 'full' protocols of increasing length and sensitivity [3]. For the purposes of the analysis here, the 'core' protocol included all the 'pencil and paper' tests and comprised 22 measures drawn from 15 tests (see Table 1). The 'full' protocol comprised the 'core' battery plus an additional 7 measures from 6 computerised tests (see Table 2). Neuropsychological tests frequently yield more than one measure. Some (i.e., the CANTAB battery of tests) yield very many, while others (i.e., SCOLP) yield essentially only one. Here, the measures employed were either those most widely validated and employed in clinical practise, or those that most selectively target the cognitive region of interest.

**Table 1:** Pattern matrix of correlations between the four principal components and individual neuropsychological tests. (correlations between -0.10 to 0.10 are not shown and correlations > 0.40 and <-0.40 are in bold). The factors explaining the greatest degree of variance was 1, followed by 2, 4 and 3.

**Neuropsychological Tests**	**Factors and cognitive domain labels**			
	**1 (VM)**	**2 (GVS)**	**3 (PS)**	**4 (NVS-M)**

AMIPB story immediate recall (IR)	**0.56**	**0.57**	-0.29	
AMIPB story delayed recall (DR)	**0.64**	**0.49**	-0.29	
CVLT word list learning trials 1–5	**0.81**		0.22	
CVLT Short delay free recall	**0.88**	-0.17	0.20	
CVLT Long delay free recall	**0.90**	-0.17	0.12	
WAIS-R digit span	-0.27	**0.63**	0.16	0.22
WAIS-R vocabulary		**0.85**		
WAIS-R block design	-0.16	0.27	0.21	**0.65**
WAIS-R arithmetic	-0.21	**0.69**	0.13	0.22
WAIS-R similarities		**0.60**	0.22	
Rey figure copy	-0.10	0.16		**0.71**
Rey figure immediate recall (IR)	0.23		-0.12	**0.85**
Rey figure delayed recall (DR)	0.26		-0.12	**0.85**
Phonemic verbal fluency		**0.40**	**0.56**	-0.14
Semantic verbal fluency	0.23	0.33	**0.44**	-0.18
RMT words	**0.49**	0.13	0.21	
RMT faces	0.34	-0.22	0.13	0.25
Boston naming (no. correct)	0.15	0.38	0.25	0.10
SCOLP	0.17	0.28	**0.70**	-0.27
SDMT written			**0.77**	0.22
SDMT oral	0.11		**0.75**	0.22

VM = verbal memory; GVS = general verbal skills; PS = processing speed; SWM = spatial working memory; NVS-M = non-verbal skills/memory; ES = executive skills. AMIPB = Adult Memory and Information Processing Battery; CVLT = California Verbal Learning Test; WAIS-R = Wechsler Adult Intelligence Scale Revised; RMT = Warrington Recognition Memory Test.

**Table 2:** Pattern matrix of correlations between the six principal components and individual neuropsychological tests. (correlations between -0.10 to 0.10 are not shown and correlations > 0.40 and <-0.40 are in bold). The factors explaining the greatest degree of variance was 1, followed by 3, 2, 5, 4 and 6.

**Neuropsychological Tests**	**Factors and cognitive domain labels**					
	**1 (VM)**	**2 (GVS)**	**3 (PS)**	**4 (NVS-M)**	**5 (SWM)**	**6 (ES)**

AMIPB story immediate recall (IR)	**0.55**	**0.61**	-0.26			
AMIPB story delayed recall (DR)	**0.63**	**0.53**	-0.26			
CVLT word list learning trials 1–5	**0.82**		0.21			
CVLT Short delay free recall	**0.87**		0.17			
CVLT Long delay free recall	**0.89**	-0.10				
WAIS-R digit span	-0.19	**0.58**	0.27	0.18		0.31
WAIS-R vocabulary		**0.79**	0.18		-0.13	-0.10
WAIS-R block design	-0.16	0.19	0.14	**0.54**	0.22	-0.15
WAIS-R arithmetic	-0.10	**0.67**	0.12		0.21	0.17
WAIS-R similarities		**0.54**	0.31			-0.19
Rey figure copy	-0.17	0.19		**0.68**		
Rey figure immediate recall (IR)	0.18		-0.13	**0.91**		
Rey figure delayed recall (DR)	0.20		-0.13	**0.92**		
Phonemic verbal fluency		0.36	**0.62**		-0.13	
Semantic verbal fluency	0.18	0.34	**0.49**		-0.19	-0.11
RMT words	**0.43**	0.16	0.24			
RMT faces	0.27	-0.20	0.23	0.34	-0.26	
Boston naming (no. correct)		0.35	0.27	0.18	-0.20	-0.28
SCOLP	0.12	0.27	**0.77**	-0.28		
SDMT written			**0.78**		0.19	
SDMT oral	0.14	-0.10	**0.75**		0.19	
CANTAB IED adjusted errors score	0.15				-0.19	**0.87**
CANTAB Spatial span		-0.11	0.13	0.12	**0.53**	
CANTAB SWM strategy score	-0.10			0.23	**-0.80**	0.13
CANTAB SWM between errors score			-0.13		**-0.79**	0.14
CANTAB SOC minimum move solutions		0.13	-0.20	0.16	**0.55**	
CANTAB PAL total trials	-0.39	0.22	-0.13	-0.15	-0.12	0.23
CANTAB RVIP 'A' prime		0.14	**0.58**		0.24	0.23

VM = verbal memory; GVS = general verbal skills; PS = processing speed; SWM = spatial working memory; NVS-M = non verbal skills/memory; ES = executive skills. AMIPB = Adult Memory and Information Processing Battery; CVLT = California Verbal Learning Test; WAIS-R = Wechsler Adult Intelligence Scale Revised; RMT = Warrington Recognition Memory Test; CANTAB = Cambridge Neuropsychological Test Automated Battery; IED = intradimensional/extradimensional set-shifting task; SWM = Spatial Working Memory task; SOC = Stockings of Cambridge task; PAL = Paired Associate Learning task; RVIP = Rapid Visual Informational Processing task.

All tests were always administered in the same sequence, beginning with pre-assessment screening, followed by the 'core' protocol through to complete the 'full' protocol. This strategy aimed to maximize the chances that a consistent minimum data set (i.e., 'core') would be obtained.

### Analyses

The N-ISAT raw data set was transformed into standardized z-scores (with a mean of 0 and a standard deviation of 1) relative to age-matched normal populations, employing standard reference manuals and materials [3]. In line with widely accepted clinical practice Individuals' test scores that fell at or below the 5^th ^percentile (equivalent to a z-score ≤ -1.645) were then identified and classified as impaired scores or 'deficits'.

Raw test scores from all patients who completed the 'core' and then 'full' protocols were included in a PCA in order to yield an empirical guide to reducing the number of dependent outcomes. *A priori *evidence suggests that while different aspects of cognitive function may be relatively independent, they are not fully orthogonal and instead co-vary. Therefore a non-orthogonal oblique rotation (promax) was undertaken on the 2 different samples of patients who completed the 'core' and the 'full' test protocols. These analyses yielded 4 and 6 factor solutions respectively. The factors were all related, to a greater or lesser extent to each cognitive test score, and in addition, to each other. Individual's z-scores on each factor were then examined in order to identify patients whose scores fell at or below the 5^th ^percentile (equivalent to a z-score ≤ -1.645). These individual factor scores were not used to estimate a CCR.

Following the classification of patients on the basis of their neuropsychometric test scores into 'impaired' and 'not-impaired' groups, we examined differences in group summary scores taken from self and relative/carer rated UK version of the Sickness Impact Profile Quality of Life questionnaire [6] using an independent samples t-test.

### Missing data

A preliminary examination of missing data has already been published [3]. In the analysis here every attempt was made to maximize the sample sizes in the calculation of a CCR; missing data were therefore re-examined. We found a substantial number of patients who failed to complete the 'core' or 'full' batteries, but for whom many measures were available. For example, of the 567 patients who passed the initial screening assessment and were seen face-to-face, all but 28 completed more than 20 measures. Here there was sufficient available test data to enable a determination of whether there was cognitive impairment, though failure to complete (or acquire) one or two measures had potentially excluded patients from a CCR based on full or core protocols. We therefore explored ways to include these cases in the overall CCR.

In addition we included missing data into the CCR analyses, where failure to complete a test had been contemporaneously classified by examiners as "SAH related" (as opposed to "SAH-unrelated") [3]. If coded "SAH-related", then the cause of the missing data was attributed to sequelae associated with the index subarachnoid haemorrhage (i.e., aphasia, sensory deficit, under 24 hour care). Failure to complete a test in this context was therefore conceptualized as a clinically meaningful endpoint, equivalent to an impoverished score on that test.

As the N-ISAT test battery had been administered in the same order it was possible to infer additional reasons for some missing data. For example the CANTAB computerized tests were routinely administered *after *patients had completed the 'core' protocol of 'pencil and paper' tests, and following a scheduled break (i.e., for lunch) in the assessment day. Therefore, if patients had already encountered difficulties coping with the 'core' battery of tests there might be a tendency for the remainder of the assessment to be abandoned (by examiner, patient or both) at this point in the day. If such missing data had been coded as 'SAH-related", the examiner would have had grounds for believing that patients' presentation up until that point in the assessment suggested they would not, or could not cope with further assessment. The structure of the assessment therefore impacted on the distribution of missing data.

### A 'sliding scale' CCR

Having transformed the raw scores on each of the 29 measures into z-scores, we then identified all those scores that fell at or below the 5^th ^percentile. All these measures were assumed, potentially, capable of sampling a relatively selective cognitive deficit (in line with the rationale underlying their inclusion in the test battery), and were therefore included in the analysis on an equivalent basis. However, with four particular pairs of test measures (i.e., AMIPB immediate and delayed recall, the Rey figure immediate and delayed recall, the CVLT short and long delay recall and the SDMT written and oral subtests) we included the mean of the two measures rather than each measure independently in the sliding scale totals. This refinement was necessary as there was a high probability that a deficit on one of these measures would be associated with a deficit on the other in the pair; indeed the subtest scores of each pair were all found to correlate significantly ≥ 0.9 (p < .001).

We then calculated the percentage of individuals with scores at ≥ 5^th ^percentile on an escalating number (i.e., 0–10) of measures. This sliding scale of cognitive impairments was calculated for the 'core' and 'full' samples, and then for all patients who had completed at least 10 measures. To further simplify these data, individuals' deficits on the sliding scale were classified into 4 categories. Where there was ≤ 1 impaired test score, patients were classified as having "no deficit"; 2 or 3 impairments were classified as a "mild" deficit, and 4, 5 or 6 impairments as a "moderate" deficit. Patients with ≥ 7 impaired test scores were classified as having a "severe" deficit.

Classifications at the two extremes of this distribution are not in our view controversial. However across the mild-moderate levels of impairment we would anticipate difficulties identifying a cut-off to differentiate those patients with a clinically or functionally significant level of impairment from those without. Clinical experience suggests that in the critical range of approximately 4–6 test deficits, determining *where and with what degree of consistency deficits lie*, can have an crucial bearing on whether a patient was likely to be functionally impaired. For example, 3 or 4 deficits selectively targeting delayed recall measures of memory might be indicative of a functionally significant amnesia, but 3 or 4 deficits distributed more randomly across the test battery might reflect nothing more than a combination of low pre-morbid ability and/or dispositional factors. These considerations were therefore incorporated into a 'cognitive domain specific CCR'.

### A cognitive domain specific CCR

In order to make allowance for the internal consistency of deficits across the test battery, we grouped measures into cognitive 'domains' based upon the six factors that emerged from the PCA analysis of the 'full' sample; reference was also made to the four factor solution that emerged from the 'core' sample. We first identified those key measures in each of the 6 PCA-derived cognitive 'domains' with loadings greater than 0.4 for that factor as follows:

1. Verbal Memory (VM): AMIPB and CVLT subtests, RMT 'words'.

2. General Verbal skills (GVS): AMIPB and all WAIS-R subtests except Block Design (n.b., phonemic verbal fluency in 4 factor solution).

3. Processing Speed (PS): SCOLP, SDMT, RVIP and verbal fluencies.

4. Non-verbal Skills/memory (NVS-M): Rey copy & delays, Block Design.

5. Spatial Working Memory (SWM): all CANTAB subtests except IED and PAL.

6. Executive skills (ES): CANTAB IED.

Clinical experience suggested that a criterion of impaired z-scores (i.e., ≤ 5th percentile) on 2 or more of these key measures (for each of the 6 PCA-derived domains), would adequately define a 'domain specific' deficit. Nevertheless two particular measures presented difficulties in this respect as they were represented across PCA-derived domains (see above). The AMIPB subtests loaded on both the VM factor and GVS factors (see Table 2), and verbal fluencies (though not reaching criterion except on the four factor solution) showed a trend (that was consistent with clinical experience), of loading on GVS and PS. Therefore, to avoid double counting domain specific deficits, we calculated a mean score for the 2 AMIPB (immediate and delayed recall) subtests and included this variable in both the GDS and VM domains. We also cross-checked all analyses with verbal fluencies placed in both the PS and GVS domains.

In order to identify a cognitively impaired individual the algorithm of 2 or more deficits in 2 or more factor-derived cognitive domains would be equivalent to identifying patients with deficits on 4 tests, but unlike the sliding scale, would exclude patients with deficits more randomly distributed across the battery, and rather target patients with greater internal consistency in the distribution of their deficits.

## Results

### Sampling

Between June 1997 and November 2001 836 patients were randomised into ISAT across the 8 N-ISAT UK centres. However, 65 died before 12-month follow-up, 198 were lost to N-ISAT follow-up and a further 197 of those 573 patients who presented for face-to-face assessment failed to complete the full neuropsychological test battery. A complete neuropsychological data set was therefore only available on 376 patients, though mRS grades were available for 828. Despite a rigorous and systematic attempt in N-ISAT to minimize and then characterize loss to follow-up [3], the non-random distribution of death and major disability was a primary trial outcome, and outside the investigators' control. The distribution of full neuropsychological data sets (or conversely, missing data) across the two arms of N-ISAT was therefore non-random, with only surviving patients 'available' for assessment, and survivors with worse outcomes less likely to attend for follow-up or complete the full test battery.

Of the 771 surviving patients theoretically available for 12-month neuropsychological follow-up, 29 (14.6%) did not attend for reasons related to the original SAH (i.e., in 24 hour hospital care), 96 (48.5%) for unknown reasons (i.e., couldn't be contacted), and 73 for reasons unrelated to SAH (36.8%).

Of the 573 patients who presented for assessment, 6 failed the pre-assessment screening protocol and 7 failed to complete > 14 test measures (for a variety of reasons). Of the remaining 560 presenting patients, 449 (80%) completed the 'core' test protocol, while 376 patients (67%) completed the 'full' test protocol. However, fully 538 patients completed at least 20 measures. Though 25.6% of the 771 patients who were theoretically available to N-ISAT were never seen face-to-face for assessment, this was primarily for reasons outside the control of N-ISAT investigators.

### Missing data

We examined the distribution of missing data coded as 'SAH-related'. And found particular difficulties with CANTAB test data. We examined all patients in the 'core' *sample *who had SAH-related missing data on CANTAB tests. From a total of 459 patients, 58 had failed to complete 1 or more CANTAB tests but fully 40 had failed to complete over half this battery of 7 tests (i.e., 4 or more tests). Further analysis indicated that the majority of these 40 patients were cognitively impaired, as judged on the basis of their 'core' protocols, and would therefore be included in the CCR on this basis without reference to their missing CANTAB data. Of 31 patients who had SAH-related missing data on 5 or more CANTAB tests, 63% were for example impaired according to the 'two domain' criterion presented in Table 3.

**Table 3:** A CCR for the 'full' and 'core' samples. A CCR for the 'full' (i.e., 6 factor solution) and 'core' (i.e., 4 factor solution) samples; where 2+ deficits or 3+ deficits (i.e., test scores < 5^th ^percentile) define a domain-specific deficit.

	**6 domains**		**4 domains**	
	
**No. of domains with deficits**	**No missing data (n = 376)**	**Including missing data (n = 459)**	**No missing data (n = 449)**	**Including missing data (n = 509)**
	***2+ measures***		***2+ measures***	
**0**	41.2	36.2	52.3	49.1
**1 or more**	58.8	63.8	47.7	50.9
**2 or more**	31.9	38.3	24.3	27.7
**3 or more**	13.3	22.9	10.2	13.8
	***3+ measures***		***3+ measures***	
**0**	54.3	47.5	67.5	63.1
**1 or more**	45.7	52.5	32.5	36.9
**2 or more**	17.3	25.7	13.1	16.1
**3 or more**	7.4	12.9	3.3	5.7

By re-coding SAH-related missing data as 'impaired' scores, the 'core' sample was increased from 449 to 509 (i.e., from 80% to 89% of available, presenting patients), and the 'full' sample from 376 to 459 (i.e., from 66% to 82% of available presenting patients). Throughout our findings detailed below, we provide estimated CCRs both with and without the inclusion of these missing data.

Those patients who were unable to attend for assessment for reasons related to the index SAH (n = 29) and those who failed the screening assessment (n = 6) or failed to complete > 14 test measures (n = 7), comprised an additional group of 42 patients who might to be included in a CCR.

#### N-ISAT neuropsychological profile

**Figure 1:**
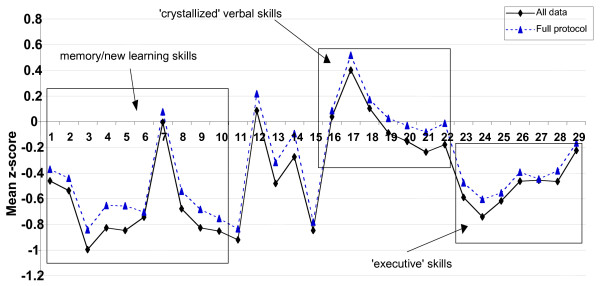
**NISAT 'z' scores on the 'full' protocol**. NISAT 'z' scores on all 29 neuropsychological measures that comprise the 'full' protocol. Scores have a mean of 0 and a standard deviation of 1, and are standardized against a normal population. 1 = AMIPB Story immediate recall (IR); 2 = AMIPB Story delayed recall (DR); 3 = CVLT word list learning trials 1–5; 4 = CVLT list IR 5 = CVLT list DR; 6 = Paired associate Learning trials 7 = RMT 'words'; 8 = RMT 'faces'; 9 = Rey figure IR; 10 = Rey figure DR; 11 = Rey figure copy; 12 = WAIS-R Block design; 13 = SDMT 'written'; 14 = SDMT 'oral'; 15 = CANTAB RVIP 'A' prime score; 16 = SCOLP; 17 = Boston naming; 18 = NART IQ; [WAIS-R 19 = Similarities; 20 = Vocabulary; 21 = Digit Span; 22 = Arithmetic]; 23 = Semantic fluency; 24 = Phonemic fluency; 25 = CANTAB IED adjusted errors score; 26 = CANTAB Spatial Working Memory (SWM) errors; 27 = CANTAB SWM strategy; 28 = CANTAB Spatial span; 29 = CANTAB Stockings Of Cambridge minimum move solutions.

Figure 1 presents the mean z-scores of the 376 patients who completed all of the 29 neuropsychological measures that comprise the 'full' protocol, and the mean z-scores on all tests for all patients (regardless of how much of the battery each individual completed).

These profiles illustrate patients' scores relative to a normal control population. The mean test scores taken from the 'full' sample of 376 patients suggested that general verbal skills and estimated pre-morbid verbal IQ (NART) clustered around the mean. On tests of verbal memory/new learning ability (i.e., AMIPB and CVLT), patients' scores, with some consistency, were shifted down by approximately 0.4 – 0.8 of a standard deviation below the mean. Similarly, on tests of executive skill (i.e., verbal fluency, IED, SWM and SOC) scores were shifted down by approximately 0.4 – 0.6 of a standard deviation. On tests of psychomotor/processing speed and spatial skills, there was less consistency in the apparent sensitivity of measures, with scores on the RVIP and Rey Copy shifted down by approximately 0.8 of a standard deviation, whereas on the SDMT and Block Design subtest (taken from WAIS-R), scores were clustered around the mean.

Figure 1 illustrates that the mean scores of all measures (i.e., regardless of the different sample sizes for each measure), are shifted down relative to the means from the constrained, smaller 'full' sample, suggesting that the 'core' sample may have for exampled tended to exclude patients with a more severe range of deficits.

#### Principal components analyses on 'full' and 'core' samples

The raw scores of 376 patients on the 28 measures that comprise the 'full' protocol (excluding NART) were entered into a principal components analysis (PCA) producing a six-factor solution, which converged after 11 iterations. The resulting pattern matrix (Table 2) shows the factor loadings for each variable on the 6 factors, after rotation. These factors may be considered empirically derived cognitive 'domains', specifically relevant to this clinical N-ISAT sample [5]. The pattern of correlations between the 28 measures suggested a labelling of the factors as follows:

#### Factor 1: cognitive domain – "Verbal Memory"

The highest loadings on this factor include measures taken from the robust neuropsychological paradigms of story recall, word list learning and word recognition that evaluate verbal memory/new learning ability.

#### Factor 2: cognitive domain – "general verbal skill"

The highest loadings on this factor include key subtests that comprise the Verbal Scale of the WAIS-R (i.e., Vocabulary, Similarities, Arithmetic, Digit Span) together with tests of verbal fluency (both phonemic and semantic). High loadings were also present on the immediate and delayed recall measures of the story recall. However, this finding was not unexpected as, though the story recall task is designed to assess verbal memory, general verbal skills underpin the ability to grasp the essential narrative structure of the task, which in turn facilitates recall performance.

#### Factor 3: cognitive domain – "processing speed"

Though the measures with the highest loadings on this factor represent a relatively heterogeneous constellation of tests (i.e., verbal fluency, SDMT, SCOLP and RVIP), they share a clearly defined commonality in so far as they comprise every test in the protocol that is performed *under time pressure*.

#### Factor 4: cognitive domain – "non-verbal skill and memory"

The highest loadings on this factor comprise measures of the copy and recall of the Rey Complex Figure, and the Block Design subtest taken from the WAIS-R. This factor is therefore a mixture of general visuo-constructive/spatial skills and non-verbal memory/new learning ability.

#### Factor 5: cognitive domain – "spatial working memory"

The highest loadings on this factor include key measures taken from tests that comprise the CANTAB battery of computerized tests (Spatial Span, Spatial Working Memory [both 'between errors' and 'strategy' scores] and The Stockings of Cambridge minimum move solutions score). Spatial Working Memory is the underlying cognitive skill common to these tasks that could influence performance on each. However, two of the 4 measures (i.e., Spatial Working Memory 'strategy' score and Stockings of Cambridge minimum move solutions), have also been conceptualized as measures of 'executive' skill, and been shown to be selectively sensitive to a variety of fronto-striatal lesions [7,8].

#### Factor 6: cognitive domain – "executive skill"

Only one measure loads on this factor, the 'adjusted error' score of the Intra-extra Dimensional Set-shifting task taken from the CANTAB battery. This task is a decomposition of the Wisconsin Card Sorting Task, and the 'adjusted error' score is a measure of the extent to which patients encounter increasing difficulties as they proceed through escalating task demands, delineated by the ability to make intra-dimensional and then extra-dimensional set-shifts. Difficulty negotiating the latter set-shift is conceptualized as an impairment in one component of executive skills.

Raw scores from the 449 patients who completed the 21 'paper and pencil' measures in the 'core' protocol (excluding NART) were then entered into a PCA. This produced a four-factor solution, which converged after 9 iterations. The resulting pattern matrix (Table 1) shows the factor loadings for each variable on these four factors after rotation. The pattern of correlations indicated that these four factors (verbal memory, general verbal skills, processing speed and non-verbal skill/memory) mapped very closely onto factors 1, 2, 3 and 4 from the six factor solution, with more or less the same key tests loading > 0.4. These findings suggested that four 'core' factors emerged consistently despite the different sample sizes (i.e., 'core' protocol 449 compared to 'full' protocol 376), and the additional CANTAB computerised test battery in the 'full' protocol. The addition of the CANTAB tests in the 'full' battery rather yielded two additional and distinct cognitive domains (i.e., factors 5 and 6 on the six factor solution).

We identified those patients with individual factor scores falling ≤ 5^th ^percentile on each factor for the six (i.e., 'full' protocol; n = 376) and four (i.e., 'core' protocol n = 449) factor solutions (see Table 4). With respect to the first four equivalent factors across each of the two solutions, Table 4 demonstrates there was a very high level of agreement (i.e., > 97%) in identifying those individuals with scores either above or below a 5^th ^percentile cut-off. We also compared agreement between the four and six factor solutions on the same sample of 376 patients, and found that this also yielded > 97% agreement in identifying those individuals who fell above and below a 5^th ^percentile cut-off.

**Table 4:** Comparison of the 4 factor and 6 factor solutions. Comparison of which patients are a < 5^th ^% percentile cut-off, on comparable factors for the 4 and 6 factor solutions. Values in the table are percentage of the sample for which both factors are available (i.e., n = 376). Numbers of patients are in brackets.

	**Factor**			
	
	**VM**	**GVS**	**PS**	**NVS-M**
**Agree: above cut-off**	93.6 (352)	92.6 (348)	93.4 (351)	94.9 (357)
**Agree: below cut-off**	4.5 (17)	5.1 (19)	4.0 (15)	3.5 (13)
**Disagreement**	1.9 (7)	2.4 (9)	2.6 (10)	1.6 (6)

### A sliding scale of CCRs

Table 5 presents a sliding scale of the percentage of patients with 0–10 (or greater) cognitive deficits (defined by a raw score falling ≤ 5th percentile relative to normative data) on cognitive measures drawn from three samples. Firstly, all 376 patients who completed the 'full' battery of measures, secondly, all 449 patients who completed the 'core' battery of measures, and finally all 560 patients who completed at least 10 measures. For each of these samples percentage deficit rates with and without 'SAH-related' missing data are included.

**Table 5:** The percentage of N-ISAT patients with cognitive deficits on an escalating number of 1–10 measures. Means are taken for the immediate and delayed recall of AMIPB, CVLT, Rey Figure, and for 2 subtests of SDMT. For the complete battery and "At least 10 measures" group, 24 measures are therefore included. For the core test group 17 measures are included. All patients have passed screening assessment.

	**FULL protocol sample**		**CORE protocol sample**		**Patients completing at least 10 measures**	
	**No missing data (n = 376)**	**Including missing data (n = 459)**	**No missing data (n = 449)**	**Including missing data (n = 509)**	**Not counting missing data (n = 560)**	**Counting missing data (n = 560)**
0	16.0	13.5	24.9	23.6	13.8	13.4
1+	84.0	86.5	75.1	76.4	86.2	86.6
2+	66.2	71.0	53.9	56.4	68.2	70.2
3+	47.6	54.0	36.5	40.5	50.0	53.0
4+	36.4	43.6	26.1	30.6	39.8	42.9
5+	27.9	35.9	18.3	22.6	30.0	34.1
6+	20.7	29.6	14.0	18.1	23.8	28.2
7+	14.6	24.2	10.2	13.8	18.2	22.7
8+	12.0	21.1	6.2	9.8	14.8	19.6
9+	8.5	17.4	3.8	6.9	11.4	16.4
10+	6.6	15.0	2.9	5.7	8.4	13.8

Table 5 illustrates that, with some consistency, including SAH-related missing data as 'impairments' increases the overall CCR *progressively*, moving up the scale toward greater severity of impairment. Thus, the more measures a patient is impaired on, the more likely they are to have SAH-related missing data. In the 'full' sample of 376 patients there was a CCR of between 20.7% to 36.4%, depending on whether the cut-off is drawn at 6 or more, or 4 or more test impairments respectively. By adding SAH-related missing data, these percentages increase up to the range 29.6% to 43.6%. A similar distribution is apparent when examining all 560 patients who completed at least 10 test measures. Here the CCR without SAH-related missing data is in the range 23.8% to 39.8% (for cut-offs of 6–4 respectively) rising to the range 28.2% to 42.9%, where missing data are included. Table 5 also illustrates that significantly fewer patient impairments were identified within the 'core' sample, where fewer tests were administered.

Table 6 presents the percentage of patients classified as having no deficit, or mild, moderate or severe deficits. The percentage of patients with 'no deficit' was very similar for the sample of 376 patients with all measures available from the 'full' protocol, and patients drawn from the much larger sample of 560 patients who only completed at least 10 measures. Approximately a third of the sample (between 31.8% to 33.8%) was essentially unimpaired cognitively in these terms. The CCR for patients classified with 'mild' and 'moderate' degrees of deficit was also very similar between these two samples, falling in the range 28.4% to 29.8% for a 'mild' deficit, and 21.6% to 21.8% for a 'moderate' deficit. These ranges applied within 1% whether SAH-related missing data was included or not. Table 6 therefore reinforces the observation that the greater the number of deficits patients have, the greater the additional 'deficit-loading' added by including SAH-related missing data. It is also apparent from Table 5 and 6 that the completed 'core' battery of 'pencil and paper' tests, though comprising a more substantial sample, yielded a much smaller CCR.

**Table 6:** The percentage of patients with increasing levels of impairment. The percentage of N-ISAT patients classified with no deficit, and 'mild, 'moderate' and 'severe' levels of impairment. No deficit = ≤ 1 impaired score on measures, a mild deficit is 2 or 3 impaired scores, a moderate deficit is 4–6 impaired scores, and a severe deficit is > 7 impaired scores

	**FULL protocol sample**		**CORE protocol sample**		**Patients completing at least 10 measures**	
	
	**No missing data (n = 376)**	**Including missing data (n = 459)**	**No missing data (n = 449)**	**Including missing data (n = 509)**	**Not counting missing data (n = 560)**	**Counting missing data (n = 560)**
No deficit	33.8	29.0	46.1	43.6	31.8	29.8
Mild deficit	29.8	27.4	27.9	25.7	28.4	27.3
Moderate deficit	21.8	19.4	15.8	16.9	21.6	20.2
Severe deficit	14.6	24.2	10.2	13.8	18.2	22.7

### A PCA-derived cognitive domain CCR

Test measures were grouped into cognitive 'domains' based upon the factors that emerged from the PCA analysis. For the purpose of illustration the percentage number of patients from the full and core samples with 3 as well as 2 deficits (i.e., on measures that load in each cognitive domain) are presented in Tables 7 and 8. The percentage CCR for each of the PCA-derived cognitive domains is presented for both the 'full' and the 'core' samples, both with and without SAH-related missing data included.

**Table 7:** Percentage of patients impaired in each of 6 cognitive domains. Numbers are the percentage of the 'full' samples who had (a) at least 2 and (b) at least 3 impaired scores in each of the 6 PCA-derived cognitive domains (n.b. domain 6 has 1 measure). Numbers in brackets are for where verbal fluency scores were placed in the in the GVS domain rather than PS domain. An impaired score is defined as < 5 percentile.

	**Cognitive domain**					
	
	**1 (VM)**	**2 (GVS)**	**3 (PS)**	**4 (NVS-M)**	**5 (SWM)**	**6 (ES)**
	***2+ measures***					

**No missing data (n = 376)**	32.2	5.3 (16.5)	21.8 (14.9)	26.3	8.8	19.4
**Including missing data (n = 459)**	35.9	9.8 (21.4)	29.6 (23.7)	28.8	17.4	21.4

	***3+ measures***					

**No missing data (n = 376)**	23.9	1.3 (5.9)	11.4 (6.4)	14.1	2.4	19.4
**Including missing data (n = 459)**	27.0	4.6 (9.8)	20.3 (15.9)	16.3	10.5	21.4

**Table 8:** Percentage of patients impaired in each of 4 cognitive domains. Numbers are the percentage of the 'core' samples who had at least 2 or at least 3 impaired scores in each of the 4 domains. Numbers in brackets are for fluency in the GVS domain rather than PS domain. An impaired score or deficit is defined as one falling < 5%ile.

	**Cognitive domain**			
	
	**1 (VM)**	**2 (GVS)**	**3 (PS)**	**4 (NVS-M)**
	***2+ measures***			
	
**No missing data (n = 449)**	32.3	6.9 (17.4)	17.4 (11.1)	28.7
**Including missing data (n = 509)**	34.8	9.4 (20.4)	23.4 (17.5)	29.3

	***3+ measures***			
	
**No missing data (n = 449)**	23.8	2.0 (6.2)	8.2 (2.4)	16.0
**Including data missing (n = 509)**	25.7	4.1 (8.6)	14.3 (9.2)	16.5

The figures in brackets (Table 7 and 8) represent the percentage of deficits in the GVS and PS domains if verbal fluency subtests are placed in the GVS rather than PS domain. Under these circumstances there is a shift in the distribution of deficits from the PS to the GVS domain, illustrating that GVS deficits are clearly infrequent when evaluated on the basis of WAIS-R verbal subtests alone. However it was found that shifting verbal fluencies into the criteria for evaluating GVS rather than the PS domain, revised the *overall *CCR downward by only 1% (see Table 3).

The percentage CCR across the first four factor domains drawn from both the 6 factor 'full' sample solution and the 4 factor 'core' sample solution were very similar (i.e., most differences < 2%), except in the case of PS. Here however, the higher number of deficits identified in the 6 factor solution can be explained by the inclusion of impaired scores from the RVIP in the PS domain (a measure that was not available to the core sample).

Tables 7 and 8 illustrate that in the N-ISAT sample deficits in memory were clearly salient, together with non-verbal skills/memory and executive skill. In contrast verbal subtests from the WAIS-R were clearly insensitive to any cognitive changes.

When the '4 factor domain' solution derived from the 'core' sample is applied to the 'full' sample of 376 patients, 84 individuals were identified as having a cognitive complication, as compared to the 120 patients identified employing the 6 factor solution. This finding suggested that 46% more patients were identified using the more extensive and sophisticated 'full' protocol of tests.

### Summary CCR

Table 3 presents CCRs, as the percentage of patients with deficits in 1, 2, or 3 PCA-derived cognitive domains (for both 'full' and 'core' samples with and without SAH-related missing data). Patients with deficits in two or more domains would be broadly equivalent to deficits on 4 or more tests, but would preferentially tend to exclude those patients with more randomly distributed deficits across tests, and place the emphasis rather upon those patients with some internal consistency in the pattern of their deficits. Thus, whereas in the sliding scale analysis there were 36.4% – 43.6% of patients from the 'full' sample with 4 or more deficits, the equivalent number of deficits grouped together into their cognitive domains yields a CCR of between 31.9% – 38.8%, (depending upon whether SAH-related missing data is included).

In our view calculating a CCR from the 'full' sample (n = 376) on the basis of 2 or more impairments on tests that load in 2 or more of 6 domains (see Table 3) represents a reasonable algorithm for identifying patients with clinically significant cognitive deficits, with an approximate balance between the risk of type 1 and type 2 errors. This method on a sub-sample of 376 patients yields a CCR of 31.9% without the inclusion of any missing data.

This CCR is however based on only 64% of those patients potentially available for inclusion from N-ISAT. Table 9 presents the distribution of additional cases that can be included in the CCR, if the criteria for their inclusion are optimised, given the availability of measures, in the following way:

**Table 9:** The N-ISAT summary CCR

**Step**	**Patient Samples**	**No of patients in each CCR group**	**No of patients with deficits in each CCR group**	**CCR for each group of additional patients (%)**	**Cumulative total number of patients**	**Cumulative CCR (%)**
**1**	**Full protocol**	376	120	31.9	376	31.9
**2**	**Core protocol**	73	25	34.2	449	32.3
**3**	**Core + missing data**	60	32	53.3	509	34.8
**4**	Completed at least 10 measures*	51	16	31.4	560	34.5
**5**	**Failed 10 measures screen**	7	7	100	567	35.3
**6**	**Failed initial screen**	6	6	100	573	36.0
**7**	**Remove those with IQ ≤ 75**	-16	-16	-	557	34.1
**8**	**Those not seen for reason related to SAH**	29	29	100	586	37.4

1. After the 'full' sample, the next most complete and consistent body of test data, is provided by those additional 73 patients who completed the 'core' (but not the full) protocol. By including these cases (now representing together with the 'full' sample, 76% of potentially available patients; i.e., 376 + 73 = 449), and identifying those patients (n = 25) with 2 or more deficits in 2 or more of 4 cognitive domains, the overall complication rate rises to 32.3%.

2. Patients with a 'core' protocol but some missing data (i.e., in the 'core' protocol) can then be included in the CCR by re-coding their missing data as impairments; 32 of these 60 patients had 2 or more deficits calculated in this way, in 2 or more cognitive domains. The CCR then rises to 34.8% based upon 509 cases (i.e., 376+73+60 = 509), which represents 87% of potentially available patients.

3. By then examining an additional 51 patients who completed at least 10 test measures, a further 16 were identified who were impaired on 5 or more tests (without consideration of their missing data). This yielded an overall CCR of 34.5% based upon 560 patients (i.e., 376+73+60+51 = 560).

4. The next set of adjustments to the overall CCR can be made by including those 7 patients who failed to complete at least 10 test measures and 6 patients who failed the pre-assessment screening battery of tests for reasons related to the index SAH. These inclusions adjusted the CCR to 36%.

5. As a correction for the risk of making a type 1 error in these analyses (i.e., including patients in a CCR who's scores on tests at < 5^th ^percentile was attributable to pre-morbid intellectual skills at < 5^th ^percentile), we excluded all those 16 patients with NART estimated pre-morbid IQs of ≤ 75. This correction reduced the CCR marginally to 34.1%.

6. Finally, if all those 29 patients who failed to attend the assessment for SAH-related reasons are included, the CCR rises to 37.4% for the entire sample (see Table 9).

In order to illustrate the functional significance of a cognitive 'complication' as detailed in Table 9, we compared the ratings of all those patients so classified with those not included in the CCR, on the UK version of the Sickness Impact Profile [6], The Functional Limitations Profile (FLP). Table 10 presents patients' and their carer/relatives' ratings on the Total, Physical and Psychosocial dimensions of the FLP. Across all FLP domains, patients with a cognitive complication had significantly higher (i.e., worse) percentage disability ratings.

**Table 10:** FLP ratings for patients with and without a cognitive complication. Self and carer/relative ratings on the FLP, for patients with and without a cognitive complication. The sample corresponds to row 7 of table 9 with patients with an IQ < 75 re-included (n = 573)

**FLP Measure**	**Cognitive Deficit**	**No Cognitive Deficit**
**Physical – self**	11.29	4.63 *
**Physical – carer**	11.66	4.76*
**Psychosocial – self**	19.59	12.18*
**Psychosocial – carer**	17.56	10.95*
**Total – self**	15.24	8.16*
**Total – carer**	14.51	7.59*

* *p *< 0.001 (independent sample t-test, equal variances not assumed)

## Discussion

As the UK based sub-study of the main ISAT trial, employing significantly different and potentially far more sensitive measures, the priority analysis in N-ISAT was to evaluate which of the two arms of the trial had preferential cognitive outcomes. This task was compromised by a body of non-random missing data in N-ISAT. On the one hand some patients did not present for assessment, while on the other, some who attended failed to complete the full test battery. A crucial aspect of these difficulties was outside the control of the investigators, related to the way in which clinical outcomes from the trial itself skewed the availability of neuropsychological data. However, feeding into this process was also the missing data that might be attributed to the length of the test battery. If increased levels of disability in one arm of the trial made it more likely that patients in that arm would not complete all the tests administered, clearly, it could be argued that a shorter test protocol might have reduced the likelihood of this occurring. In this sense, one of the unique strengths of N-ISAT – the sophistication of the chosen neuropsychological battery, was also a potential weakness.

The comprehensive nature of N-ISAT neuropsychological test protocol may have increased the likelihood of missing data, however we believe that by virtue of the controlled methods that must be applied in their face-to-face acquisition, all neuropsychological data, are routinely vulnerable in this respect. Further, in NISAT fully 25% of patients were 'lost to follow-up' in addition to the 34% who attended but failed to complete the full protocol (including from 1 to x missing data points); this suggests that the problem of patients' failure to attend may be nearly as salient as ensuring that the assessment is completed once they do. In any event, the purpose of a test battery must be carefully considered before implementation. For example, a battery designed to simply identify patients with a relatively severe level of overall (i.e., non-specific) cognitive impairment could be much briefer than the more extensive batteries that are necessary to pick up milder or more subtle cognitive impairment, (or enable the fractionation of brain-behaviour relationships). As mortality and morbidity rates improve across interventional medicine, it is likely that the distribution of less severe or more subtle cognitive impairment may become increasingly important in differentiating preferred outcomes [9]. Detecting these more subtle deficits is likely to require more sophisticated and possibly longer test batteries. Our analysis suggested for example that within our sample of 376 patients (with complete data sets), the 'full' N-ISAT test protocol identified approximately 43% more patients as being significantly impaired than the more limited 'core' protocol.

For the purpose of refining the representation of multiple endpoints and accommodating the problems of non-random missing data, we have described a systematic process by which individual patients with more or less complete neuropsychological profiles, might be classified as having acquired a cognitive 'complication'. The CCR we have derived should properly be viewed as an estimate as there is no available or agreed external validation criterion against which it can be referenced. Nevertheless the process by which we have arrived at this estimate is in our view relatively straightforward, as it is based upon well-established analyses (i.e., PCA) and principles of clinical practice (the 5^th ^percentile cut-off for 'impaired' z-scores). On the basis of clinical experience and expertise we have also introduced into this framework a number of adjustments and corrections to refine the estimate. Where consensus is less secure, we have relied upon transparency to accommodate a range of opinion. Thus for example we present CCR estimates with and without SAH-related missing data incorporated, and illustrate the consequences for cognitive domains based upon different numbers of impaired test scores, and a CCR based upon different numbers of 'impaired' cognitive domains. Tables 3, 6, 7 and 8 therefore might be seen as presenting a range of 'confidence intervals' for a CCR, depending upon what assumptions are made about the significance of different distributions of impaired test scores.

In clinical practice, Neuropsychologists are required routinely, to make dichotomous judgements about whether or not an individual is suffering from a functionally significant level of cognitive impairment. These judgements are characteristically based upon both quantitative and qualitative information, and negotiated with the patient. Thus, in addition to a profile of psychometric test scores, a patient's clinical presentation, medical history, 1^st ^or 3^rd ^party subjective reports of sequelae, the results from questionnaires or other clinical scales, might all be employed to provide a context for interpretation. However in contrast the identification of individuals with a cognitive 'complication' (i.e., that are assumed to have a 'functionally significant' level of cognitive impairment) in a trial the size of N-ISAT is, in the first instance, compelled to proceed on the basis of profile of psychometric test scores alone – a profile that is only one piece in the complex puzzle that is overall clinical outcome.

Though psychometric tests have the potential to be exquisitely sensitive measures of outcome following brain disease or injury, the functional significance of cognitive impairment may be more or less salient, depending upon a number of other factors. These might include for example, patients' subjective awareness of their impairment, their pre-morbid work or social status, or the presence of other neurological or psychological sequelae. It could be argued that the information that is crucial from the point of view of clinicians, trialists and most importantly the patients themselves is whether the impairment is functionally significant. In order to unravel and understand the complex associations between the different perspectives on 'outcome' from the neuropsychometric through the subjective to the functional, criteria for classifications of cognitive 'impairment' from a psychometric point of view must first be determined. The validity (or functional significance) of these classifications can then be tested and explored with reference to other contemporaneous measures of outcome. In N-ISAT, for example there are interview, HQOL questionnaire and mRS outcome data available for this purpose, though it is beyond the scope of this paper to explore these relationships fully.

## Conclusion

The evaluation of neuropsychological outcome in a large multi-centre RCT involves a number of unique organizational and methodological challenges. The measures employed may need sufficient range and sensitivity to sample adequately deficits that could be associated with a potentially heterogeneous distribution of brain lesions. However, an assessment of this length and sophistication yields potentially, a number of difficulties both in terms of its acquisition and subsequent analysis, including problems associated with having a large number of endpoints contaminated by missing data.

We have demonstrated in the N-ISAT data set that such problems can be met by exploring transparently, ways in which 29 neuropsychological outcomes can be classified and summarised. Establishing individual clinical outcomes comprising either the presence or absence of a 'cognitive complication' required an algorithm based upon the establishment of 2 'cut-offs'. Firstly a score below which individuals' performance on each measure could be said to be 'impaired', and secondly the number and/or distribution of 'impaired' scores identified in this way, required to classify and individual as having a clinically significant cognitive 'complication'. Placing the first of these cut-offs at the 5^th ^percentile is in our view uncontroversial. However we would anticipate a greater range of opinion regarding where the second cut-off should fall. We have therefore presented a 'sliding scale' of cognitive impairment. We further refined this analysis in 2 ways. Firstly, by classifying patients according to the number of acquired deficits, and secondly by taking into account the distribution of deficits, in terms of a PCA-derived set of cognitive domains (and the most representative measures included therein). Throughout these analyses we have presented the estimated CCRs, both with and without consideration for missing data of potential clinical significance. Employing these methods we have estimated an overall CCR in N-ISAT of 34.1% for all those 557 patients who were seen face-to-face for assessment, and found that this group of patients had significantly more disability on a Health Related Quality of Life questionnaire, than patients classified as having no functionally significant cognitive deficits.

## Competing interests

The author(s) declare that they have no competing interests.

## Authors' contributions

RBS and KC participated in the grant application; conception and design of the trial; acquisition, analysis and interpretation of data; writing and revising the manuscript and are responsible for the integrity of the work as a whole from inception to the finished article. AL participated in the analysis and interpretation of data, and revision of the draft. FE participated in the analysis and interpretation of data. All authors read and approved the final manuscript.
